# Organogel of Acai Oil in Cosmetics: Microstructure, Stability, Rheology and Mechanical Properties

**DOI:** 10.3390/gels9020150

**Published:** 2023-02-10

**Authors:** Suellen Christtine da Costa Sanches, Maria Inês Ré, José Otávio Carréra Silva-Júnior, Roseane Maria Ribeiro-Costa

**Affiliations:** 1 Laboratory of Pharmaceutical Nanotechnology, College of Pharmacy, Federal University of Pará, Belém 66075-110, Brazil; 2 IMT Mines Albi-Carmaux, CNRS UMR 5302, Centre RAPSODEE, Campus Jarlard, Université de Toulouse, CEDEX 09, 81013 Albi, France; 3 Laboratory R&D Pharmaceutical and Cosmetic, Faculty of Pharmaceutical Sciences, Federal University of Pará, Belém 66075-110, Brazil

**Keywords:** acai oil, 12-hydroxystearic acid, hyaluronic acid, organogel

## Abstract

Organogel (OG) is a semi-solid material composed of gelling molecules organized in the presence of an appropriate organic solvent, through physical or chemical interactions, in a continuous net. This investigation aimed at preparing and characterizing an organogel from acai oil with hyaluronic acid (HA) structured by 12-hydroxystearic acid (12-HSA), aiming at topical anti-aging application. Organogels containing or not containing HA were analyzed by Fourier-transform Infrared Spectroscopy, polarized light optical microscopy, thermal analysis, texture analysis, rheology, HA quantification and oxidative stability. The organogel containing hyaluronic acid (OG + HA) has a spherulitic texture morphology with a net-like structure and absorption bands that evidenced the presence of HA in the three-dimensional net of organogel. The thermal analysis confirmed the gelation and the insertion of HA, as well as a good thermal stability, which is also confirmed by the study of oxidative stability carried out under different temperature conditions for 90 days. The texture and rheology studies indicated a viscoelastic behavior. HA quantification shows the efficiency of the HA cross-linking process in the three-dimensional net of organogel with 11.22 µg/mL for cross-linked HA. Thus, it is concluded that OG + HA shows potentially promising physicochemical characteristics for the development of a cosmetic system.

## 1. Introduction

Organogels (OG) are viscoelastic materials composed of structurants and a nonpolar liquid phase (natural oil), forming semi-solid systems, in which an oil phase is immobilized by a self-sustaining three-dimensional net of the structuring agent [1,2]. They are produced from the mechanism of trapping the oil molecules in the three-dimensional fibrous nets formed during their self-assembly, so the structuring or gelling agents are heated with the organic compound and solubilized in it. Then, they are cooled to below a gelation transition temperature. The choice of gelling agent can develop different types of organogels, which can be classified by the size of their oil-structuring molecules into high or low molecular weight. High molecular weight organogelators (HMOGs) are mainly polymers structured by an organic solvent forming physical or chemical interactions between filaments, which results in a supramolecular net. Low molecular weight organogelators (LMOGs) can be further subdivided by the oil structuring method, through the formation of a three-dimensional net of crystalline particles or a space-filling fibrillar net [3].

OG obtained by LMWGA are frequently used in cosmetology for having the advantage of great flexibility of use associated with the ability to gel even at low concentrations of the gelling agent, typically at 2% (*m*/*m*) [3,4,5,6,7]. Furthermore, low molecular weight organogelators have a number of advantages in the application of organogels. The solid fibrillar matrix of the organogels improves the mechanical properties, resulting in a better encapsulation rate of lipophilic or amphiphilic molecules, with homogeneous distribution within the structural structure of the particles. The active ingredient, which can be in a liquid state, can be gelled directly and remain the main component of the formulation; the consistency can be easily controlled depending on the concentration of the organogelator [3]. 12-hydroxystearic acid (12-HSA) is the best known among LMWGA. It is widely used in cosmetic preparations and it is obtained from hydrogenated castor oil [8,9].

The oil plays an important role in the gelation process by LMWGA, as the interactions between the structuring molecules with organogelator compete with organogelator–organogeller interactions, and, in the synthesis of organogels, it has been shown that oils rich in long-chain monounsaturated fatty acids and polyunsaturated fatty acids promote self-assembly in polymorphic nets [3,10,11]. The nutraceutical properties of natural Amazonian oils are not limited only to their lipid composition, but also include the presence of other substances, called unsaponifiable matter, which have important biological properties, which is why they are used as humectants, emollients, emulsifiers, and viscosity adjusters in the cosmetics industry; in addition, bioactive substances can be found in Amazonian vegetable oils, such as some liposoluble vitamins, which have a protective action against the evolution of natural degenerative processes that lead to diseases and premature aging. These antioxidant substances are extremely important to reduce the harmful effects of free radicals generated by oxidative processes in our metabolism [3].

Among the vegetable oils, acai oil stands out (Euterpe oleracea Martins) as an original cosmetic ingredient originated in the Amazon rainforest which, in addition to being part of a carrier system, may offer countless benefits for the maintenance of skin balance. Its structure is mainly composed of anthocyanins, flavonoids, and carotenoids that are widely used by the cosmetics industry as antioxidants to prevent skin aging due to promoting cellular metabolism and reducing inflammatory processes. Additionally, its lipid composition may be involved in reducing the inflammatory process and nociception. Furthermore, its high concentration of polyphenols has a considerable capacity to eliminate superoxide and peroxyl radicals, which suggests that acai has anti-aging properties which can be used in the manufacture of cosmetic bioproducts [3,10,11]. In the pharmaceutical field, organogels can carry both hydrophilic and lipophilic actives, especially hydrophilic substances for topical (dermal) or transdermal administration, but they have also been evaluated in oral, rectal, and parenteral administration [1].

Highly hydrophilic and a natural moisturizing factor substance, hyaluronic acid (HA) is used in most rejuvenation treatments since, due to its molecular characteristics, gelatinous and thick consistency, elasticity and self-hydration degree, it acts as a space filler. It contributes to improve the structure and elasticity of skin by removing wrinkles, enhancing and restoring facial volume, creating lip volume, smoothing expression lines, fibroblast depletion and scarring, as well as providing facial rejuvenation [12,13,14,15]. Moreover, it has antioxidant effects as it acts as a free radical scavenger, which increases skin protection from ultraviolet radiation and helps to increase tissue repair capacity. In epidermis, they stimulate the endogenous high molecular HA, which has a positive effect on hydration. At dermal level, HA decreases the creation of pro-inflammatory interleukin cells, which are responsible for generating radicals capable of damaging both skin cell components and the extracellular matrix itself [16,17,18].

Skin is the main organ of the human body that acts as the first obstacle for microbial invasion and dehydration, besides physical and chemical damage. Therefore, skin problems, such as nasolabial folds, wrinkles, dry skin, collagen depletion, and premature aging can be treated by using HA as a cosmeceutical. HA has been used in a wide range of cosmetic formulations due to its strong water holding capacity. HA can also be used to maintain skin turgidity, moisture, and elasticity. HA has significant nutricosmetic and cosmetic efficacy in resolving different skin defects, such as premature skin aging, nasolabial folds, and wrinkles. Aiming at studying nutricosmetic and cosmetic effects, HA has been used in different forms, for example: creams, serums, gels, lotions, intradermal filler injections, and facial fillers. The nutricosmetic and cosmetic effects of HA have been linked to its ability to introduce facial rejuvenation, collagen stimulation, and tissue augmentation [16,18,19].

César et al. [20] evaluated the physicochemical characteristics of four emulsions based on organogel supplemented or not with sunflower, macadamia, or olive oils. It demonstrated that the vegetable oils added to the formulation did not change the pseudoplastic rheological behavior, but increased the hysteresis area and reduced the shear work of the formulations. In addition, sunflower seed oil increased the consistency index and all texture parameters while macadamia oil reduced the firmness and consistency of the organogel. Besides that, the cosmetic formulation based on sunflower seed had the highest score in the sensory evaluation. Yang et al. [21] investigated the application of pluronic lecithin organogel (PLO gel) in cosmetics as a topical drug delivery system, and showed that PLO gel (hydrogenated lecithin 5,0%, PEG-400 20.0%, and poloxamer 407 15.0%) can be used in an active substance delivery system, as well as one of the ingredients in cosmetic formulations. Cui et al. [22] demonstrated a multifunctional injectable hydrogel prepared with HA, dopamine, and carboxymethyl chitosan (CMC/HA-DA) to repair skin lesions; this CMC/HA-DA hydrogel has excellent adhesive, antioxidant, and hemostatic properties which strongly contributed to promote the repair of skin lesions. Zhang et al. [23] designed a hydrogel loaded with alginate, HA, and polyllysine (PLL) to obtain anti-biofouling and antioxidant properties; after loading curcumin and epigallocatechin gallate (EGCG), hydrogel drug delivery can significantly weaken the development of irradiation-induced skin lesions.

In the search for increasingly safe, biocompatible, and effective cosmetic formulations, a proposal is the combination of vegetable oils with antioxidant characteristics, such as acai oil, to other preferably natural actives, such as HA, in order to provide different functions and associated effects, such as preventing premature aging, providing elasticity, firmness, and hydration, among other skin benefits [24,25]. In this context, this research investigates the physicochemical parameters in the use of acai oil as an ingredient for the preparation of organogels and the incorporation of hyaluronic acid into acai oil-based organogel as a proposed system for cosmetic use.

## 2. Results and Discussion

### 2.1. Fourier-Transform Infrared Spectroscopy (FTIR)

**Figure 1:**
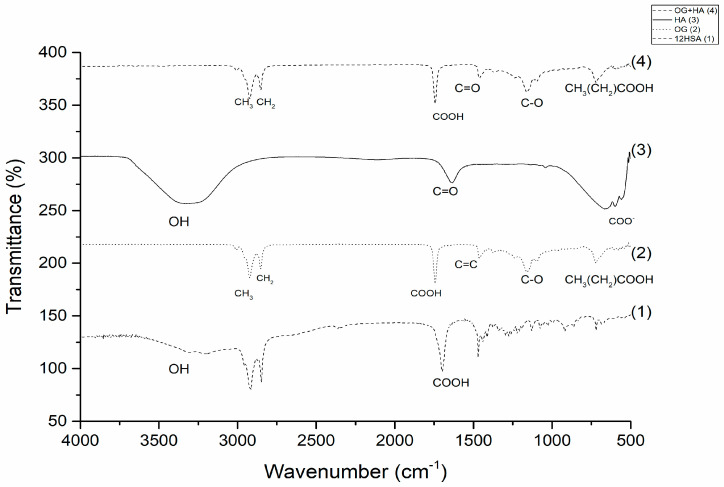
FTIR spectrum of 12HSA, OG, AH, and OG + HA submitted to a resolution of 2 cm^−1^ and a range of 4000 to 400 cm^−1^.

The observation of spectral bands (Figure 1) assisted in the evaluation of the acai oil gelling process through the structuring agent 12 hydroxystearic, forming the organogel without the active (OG). The structure of 12HSA is characterized by the presence of signals at 3200 cm^−1^ related to the hydroxyl group (-OH), and carboxylic acid groups (-COOH) at a wavelength of 1700 cm^−1^ [26,27,28,29,30].

Studies indicate that the gelation of acai oil occurs from 12-HSA through the dimerization of carboxylic acid monomers, and the longitudinal growth occurs via hydrogen bonding between the hydroxyl groups. With this, in the OG spectrum it is possible to observe the absorption bands characteristic of 12HSA at 1700 cm^−1^, which corresponds to carboxylic acid groups (-COOH), and a decrease in the hydrogen bond between hydroxyl (-OH) groups happens. In addition, the characteristic bands of acai oil are observed between 3000 cm^−1^ (methyl (-CH_3_)), 2750 cm^−1^ (methylenic (-CH_2_)), 1500 cm^−1^ (carbon double (C=C)), 1000 cm^−1^ (acyl group (C-O)), and 750 cm^−1^ (aliphatic fatty acid chains (CH_3_(CH_2_)COOH)) bands [28,31].

After evaluating the OG structure, the spectral signals of organogel containing hyaluronic acid (OG + HA) were verified first by observing the spectrum of the HA. Hyaluronic acid (Figure 1) shows a broad absorption of hydroxyl elongation (OH) at 3400 cm^−1^ and the carbonyl (C=O) signals of amide I at 1615 cm^−1^ and the length below 1000 cm^−1^ corresponds to the ester group linked to the aromatic ring [32].

The aforementioned peaks are all observed in OG + HA formulation (Figure 1); bands at 1700 cm^−1^ (-COOH) confirm the OG gelation process; the immobilization of HA in the crosslinked structure is confirmed by the visualization of the carbonyl signal linked to amide I (1615 cm^−1^), indicating that a peak shift occurs in the organogel when compared to the free active since the crosslinking reaction was superimposed on the NH peak of HA amide groups, which suggests that the drug does not interrupt the cross-linking structure of organogel and interacts with OH chains of the cross-linking agent. Furthermore, the presence of acai oil is visualized by the bands 3000 cm^−1^ (-CH_3_), 2750 cm^−1^ (-CH_2_), 1000 cm^−1^ (C-O), and 750 cm^−1^ (CH_3_(CH_2_)COOH) [33]. The spectra evaluated confirmed the formation of OG and OG + HA through the characteristic signals of each component in the formulation.

### 2.2. Polarized Light Optical Microscopy (PLOM)

**Figure 2:**
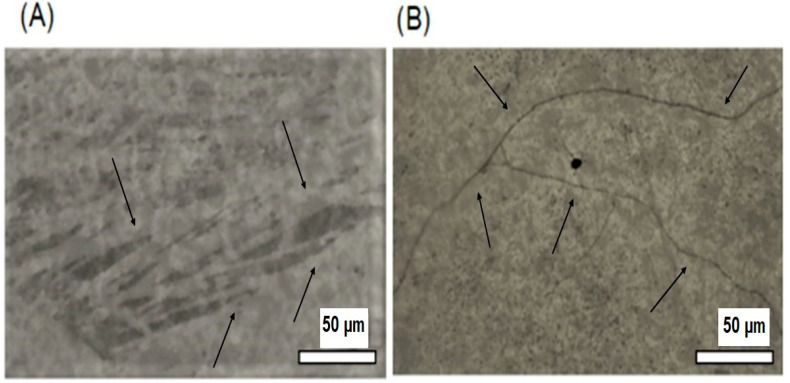
Microphotographs of OG (**A**) and OG + HA (**B**). Magnification at 50×.

Figure 2A,B present, respectively, the microphotographs of OG and OG + HA organogels obtained by PLOM.

The microstructure analysis by optical microscopy under polarized light is widely used in oils and fats, as it allows observing the typical net formed by crystals. The technique exploits the high contrast between the solid material that refracts light and the liquid phase that does not. Therefore, it allows the observation of structured crystalline materials linked to a dense tangle of fibers even at low concentrations [34], which helps to understand the macroscopic characteristics of the organogel [35].

The OG morphology visualized in the MOLP (Figure 2A) after irradiation by visible light exhibits a spherulitic texture due to the presence of crystals in the form of needles, which forms a net-like structure [26]. In OG + HA (Figure 2B), we can observe long twisted fibers that have segmented areas of brightness and dark zones that relate to the distorted nature of the gellant ribbons [36,37]. The presence of crystals and twisted fibers may indicate the occurrence of a lamellar organization characteristic of a dispersion of multilamellar vesicles in an isotropic solution with a crystal net typical of organogels, according to literature [38]. These crystals unite and lead to the crystallization of the gelling agent in the form of fibers, which forms the three-dimensional net that gives the macroscopic characteristics of the organogel [26]. Moreover, the microphotography of OG + HA corroborates with the FTIR analysis in which the active does not disrupt the cross-linked structure of the organogel.

Organogels with lamellar arrangement molecules in a fibrillar self-assembled net with the presence of oiled (isotropic) streaks and some cross-linking [26,39] have been reported in literature for different organic materials and structuring agents. Abraham et al. [40] established that isomers with hydroxyl beyond position 6 promoted crystal growth along the secondary axis of the molecule, which leads to crystallization of 12HSA as fibers. Toro-Vasquez et al. [41] showed that 12HSA gel nets were in the form of fibrillar fibers and spherulites. Hence, PLOM photographs found that there is indeed a cross-linked organization formed by crystals and twisted fibers in the studied organogels, OG and OG + HA.

### 2.3. Thermal Analysis

**Figure 3:**
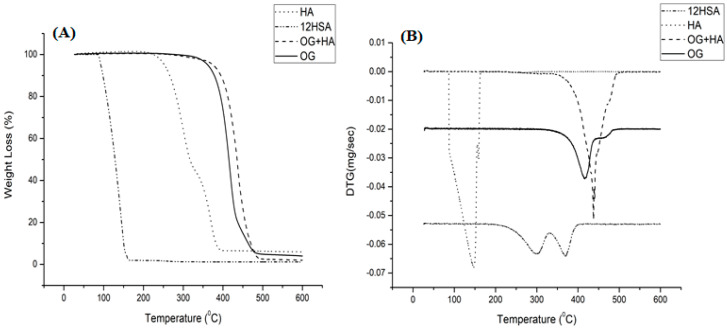
TG (**A**) DTG (**B**) curve of HA, 12HT, OG and OG + HA in N_2_ atmosphere (50 mL/min) and 10 °C/min heating, in a temperature range from 25 to 600 °C.

In the analysis of TG/DTG curve shown in Figure 3A,B, 12-HSA demonstrated two thermal events that may be correlated with the oxidation of molecules that compose this saturated fatty acid. The first one takes place around 266.01–312.43 °C, with a 52.91% mass loss, which indicates the evaporation of volatile substances. The second one takes place at approximately 359.81–381.96 °C, with a 41.10% mass loss due to the degradation of its short chains when subjected to high temperatures. Hyaluronic acid demonstrated the evaporation of water and the breaking of the ester bond present in the molecule in a single thermal event that occurs around 113.10–154.22 °C, with a 97.52% mass loss. OG illustrated two degradation events. The first, at 395.75–436.81 °C (89.89%), is responsible for decomposition of most of the substance with evaporation of water molecules. The second, at 416–499 °C (10%), represents the end of the degradation process with the breakage of unsaturated chains. OG + HA exhibited a high stability thermal profile since it increased the stability of its constituents at high temperatures, which presents only one thermal event around 413.25–457.02 °C, with a 97.69% mass loss. It corresponds to the evaporation of water molecules and the breakage of short unsaturated chains and ester bond [42,43,44].

**Figure 4:**
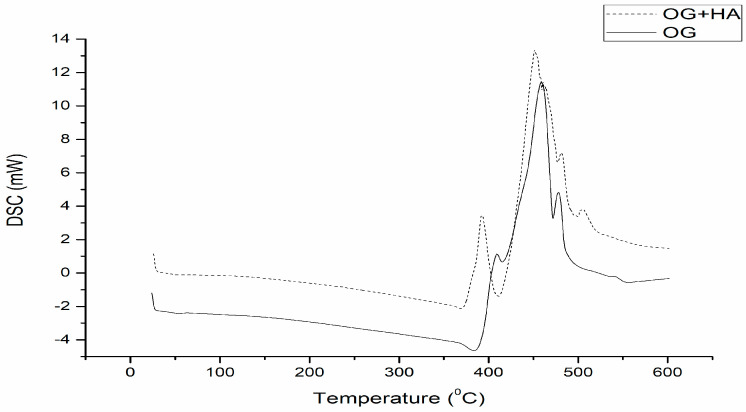
Differential exploratory calorimetry curve of OG and OG + HA in N_2_ atmosphere (50 mL/min) and 10 °C/min heating, over a temperature range from 25 to 550 °C.

DSC analysis (Figure 4) of OG showed a thermal event (422.75–473.47 °C) with an enthalpy of 842.36 j/g, which may be correlated with crystallization, as literature describes that the structuring agent promotes gelation at temperatures corresponding to concentration and thermal stability of the organic compound used. In OG + HA DSC analysis, two exothermic events were observed. The first (394.40–448.54 °C; 350.90 j/g) indicates the beginning of gelation with intermolecular interaction of water to form a cross-link [45]. Furthermore, the gel crystallization phenomenon is completed in the second event (458.37–502.92 °C; 700.52 j/g), which indicates that, with the insertion of the active, the gelation process needs to occur in two stages for its proper incorporation [46]. The acai oil-based organogels have higher crystallization points compared to other organogels composed of other vegetable oils, such as soybean oil [47].

### 2.4. Texture Profile Analysis

**Figure 5:**
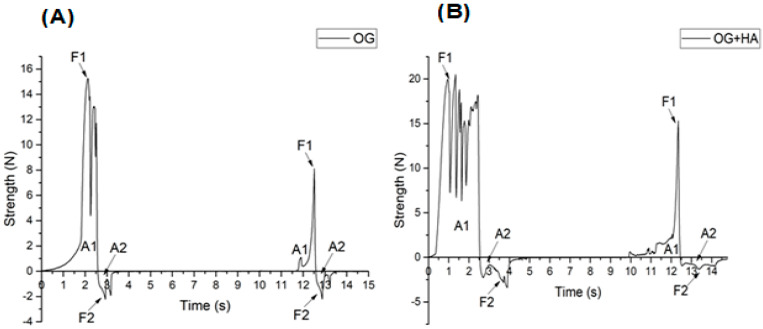
Strength over time in a double compression test of OG (**A**) and OG + HA (**B**).

Hardness can be classified as the maximum force recorded in the compression of the sample. Spreadability refers to the tendency of the sample to recover its original shape after being subjected to deformation. Adhesiveness corresponds to a negative force due to the work required to overcome the force of attraction between the sample and the probe. Gumminess relates to the force required to disintegrate a semi-solid sample [48]. In Figure 5A,B, a profile of two force cycles over time is presented. In this context, the maximum force is calculated initially by observing a rupture force. After a period of spreadability, a maximum force is visualized followed by disintegration. Then, an adhesion phenomenon (recorded as negative force) is seen. It happened due to the viscosity of the material when the probe was removed from the sample.

The hardness parameter stands out in this evaluation, and it is possible to observe the two consistency cycles in Figure 5A. In the first, OG demonstrated maximum force around 15 N. In the second moment, the value changed to 8 N; OG + HA (Figure 5B) also presented two cycles, but with a maximum strength superior to the previous organogel, which is around 20 N in the first peak and 15 N in the second peak. These results show that the samples obtained higher strength at 1 N, which indicates the strength of the crystalline net formed at this concentration. Then, it extends the possibility of application of these samples in pharmaceutical and cosmetic formulations [49].

Double compression tests are useful for evaluating changes in the physical behavior of organogels [50,51]. In this case, it was possible to observe that the insertion of the active ingredient promoted an increase in the mechanical resistance of the organogel, which is most likely caused by an increase in the organization of the organogel net [8,52,53]. Furthermore, the values found show the ability of the structuring agent to gel the liquid lipid matrix, which results in the obtainment of a relatively firm organogel with viable topical application.

### 2.5. Rheology

**Figure 6:**
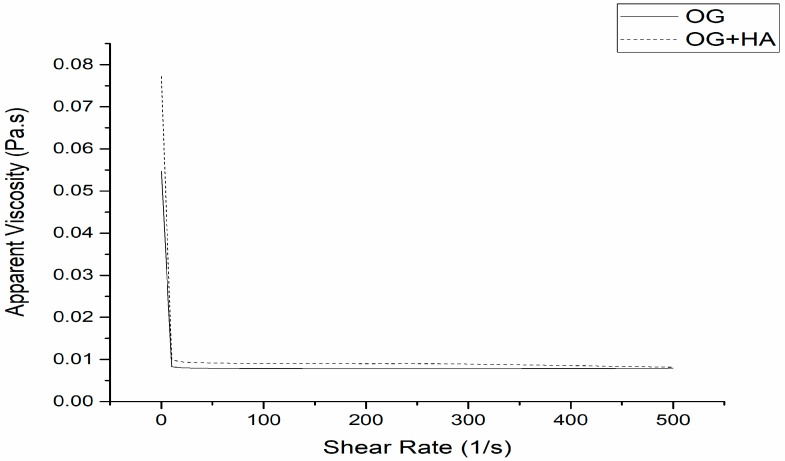
Apparent viscosity of OG and OG + HA.

The flow curve analysis allows classifying the systems as Newtonian fluids. That is, when the viscosity remains constant and there is variation of the shear rate, which is influenced only by changes in temperature and composition; or non-Newtonian fluids, which are characterized by interactions between their components that affect the deformation speed caused by external forces, which results in non-proportionality between the rate and shear stress [54]. Figure 6 shows that the organogels studied present a non-Newtonian profile since the apparent viscosity decreases as the shear rate increases, which is a characteristic of a viscoelastic material behavior, such as organogels obtained through waxes and oils. Obtaining formulations for topical use with a viscoelastic character is highly desired as they deform during application. In other words, they become more fluid, which facilitates spreading and recovering the initial viscosity at the time of application. It prevents the product drains and allows an even distribution over the skin [55,56,57].

**Figure 7:**
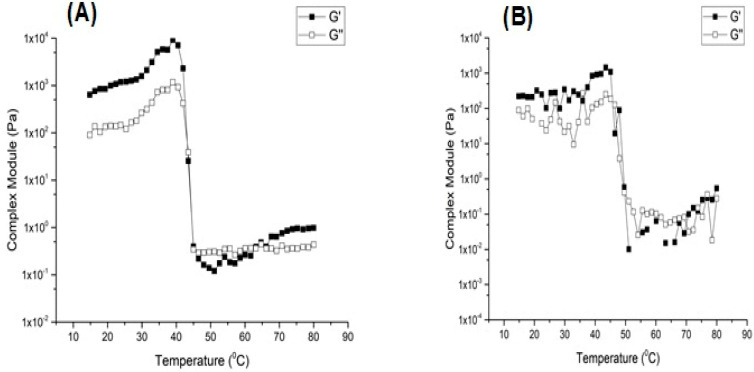
Complex module of OG (**A**) and OG + HA (**B**).

Figure 7A,B demonstrates the temperature scan analysis by evaluating the response of the material to increasing the temperature. Therefore, information about viscosity and elasticity of the gel can be obtained. The G′ parameter indicates the material’s ability to store energy, and G″ refers to the material’s ability to dissipate energy. If the G″ parameter is greater than G′, there is viscous liquid behavior, such as dispersions. If G′ is greater than G″, the system behaves like an elastic solid, such as a gel. However, if G′ is equal to G″, the material is in transition [7,58]. Thus, it was observed, in Figure 7A,B, that the evaluated organogels showed predominantly elastic behavior since both OG and OG + HA showed G′ values higher than G″. It did not cross the curves and presented a flow rate when subjected to temperature variation, which corroborates with the previous result that shows the viscoelastic characteristic of organoleptic [54]. The results are consistent with literature. For example, Lupi et al. [59] demonstrated that organogels with beeswax had a high value of G′ in relation to G″, which is associated with the strength of the net formed between the oil and the structuring agent, which increases the strength and firmness of the gels.

### 2.6. Quantification of Hyaluronic Acid

Hyaluronic acid was quantified by spectrophotometry in the ultraviolet region through a colorimetric technique by using the carbazole indicator. The reaction of uronic acids with carbazole is the most satisfactory method for estimating the amount of uronic acids in chromatographic fractions, but it requires two hours for complete color development. Besides, in the presence of certain components, the color is partially influenced by salts. In addition, the coloration is unstable and sensitive to heating, to dilution with water, and to impurities in the reagents or samples [60].

The cited difficulties of the method added borate ions to concentrated sulfuric acid, which amplified the coloration in the reaction of carbazole with uronic acid. In this way, the sensitivity of the technique is increased, and the coloration appears immediately. Besides presenting greater stability, there is great reproducibility and reduction in the interference of chloride ions and oxidants, which makes the method faster and more quantitative [61]. The total HA concentration in organogel consists of the HA present in the gel phase (insoluble) and in the fluid phase (soluble). Many manufacturers use a fluid phase to facilitate the application of the product. The soluble fluid phase consists of non-cross-linked HA. Therefore, it is easily metabolized, and it does not contribute to the effectiveness and duration of the product at the application site. Only the cross-linked HA in the gel resists enzymatic and radical degradation, which contributes to the effectiveness of the product. In this study, the presence of cross-linked HA in organogel was evaluated [62,63].

The principle of this method consists of a complete hydrolysis of HA, a conversion of released monosaccharides into furfural derivatives (under highly acidic conditions and a temperature of 95 °C) and a chemical complexation with a carbazole reagent to form a violet chromophore that can be analyzed by spectrophotometry [64]. With the result achieved (11.22 µg/mL) after obtaining the equation of a straight line, we can observe the efficiency of the HA cross-linking process in the three-dimensional net of organogel.

### 2.7. Organogel Oxidative Stability

The oxidative stability study under three different temperature conditions for 90 days evaluated the organoleptic parameters (coloration, odor, texture, and phase separation), pH, and active concentration of the OG + HA formulation. During the period of analysis, no significant differences (*p* > 0.0001) were observed in the parameters in relation to time at room temperature (Table 1), refrigeration temperature (Table 2), and kiln temperature (Table 3).

The formation of organogels, although quite simple, requires some care, especially with respect to the evaluation of organogel formation and phase separation under controlled storage conditions. These evaluations allow observing the minimum concentrations for the formation of organogels, as well as the stability of this material for longer periods, which allows to discard samples and materials that do not form stable organogels [65]. The evaluation of stability is important as the formulations, when stored, may show signs of destabilization, such as sedimentation, phase separation and inactivation of the drug delivered [66], which indicates destruction of its three-dimensional net structure [67].

Initially, the formulation presented itself as a soft gel with a viscous semi-solid texture, besides being homogeneous and presenting a typical color of acai oil (green), as well as a characteristic odor. After 90 days of analysis, the samples at room temperature (Table 1), refrigeration temperature (Table 2), and kiln temperature (Table 3) maintained their initial organoleptic characteristics throughout the experiment. At the end of 90 days, the pH of the stored samples showed a range between 5.5 and 6.9. It demonstrates that the temperature did not influence the chemical stability of the formulation components and the active ingredient since the samples remained with a pH compatible with the pH of skin (4.5–7) [66,68]. The drug concentration in the formulation maintained its value around 5.19–9.08 µg/mL (52.42–91.41%), which indicates the stability of HA under the different conditions analyzed.

Thus, no signs of instability were observed under the conditions evaluated, such as phase separation, precipitation of drug particles, nor were there sudden changes in pH that could cause skin irritation. In this sense, it shows that the formulation is capable of withstanding temperature variations during its storage period. The conservation of the active ingredient in the formulation, as well as the other results, show the chemical, physical, and therapeutic integrity of the drug, besides the pharmaceutical form under the influence of environmental factors as a function of time [69].

The results found are similar to the literature data, as Singh et al. [70] suggested that organogels formed from sorbitan monopalmitate and castor oil were stable for long periods and, therefore, can be used for the development of formulations for commercial use. Zahi et al. [71] showed that the stability of D-limonene organogel nanoemulsion was also investigated under two different temperatures (4 and 28 °C) during two weeks of storage. The results showed good stability of the formulations, which must have been materialized due to the incorporation of D-limonene in the organogel before homogenization.

## 3. Conclusions

This research shows that acai oil presents itself as a potential raw material for the development of organogels, as an organogel based on acai oil can be considered a new versatile formulation. This is because it is possible to modify the structuring agent, the active principle, and their respective concentrations in order to optimize their effects or according to the purpose of the study, which opens up new perspectives for cosmetic or pharmaceutical use.

The organogel of acai oil containing hyaluronic acid presents physicochemical characteristics suitable for a cosmetic formulation, such as low-cost composition, preparation that does not require complex equipment, thermal stability during production, and low concentration of inputs, which reduces the sensorial perception during consumption, among other advantages that facilitate its formulation and avoid rejection by the final consumer. Thus, the studied parameters demonstrate that the acai oil-based organogel containing hyaluronic acid has potential for the development of a cosmetic system.

## 4. Materials and Methods

### 4.1. Materials

Acai oil (Lot: H2004410) was obtained from the company BERACA natural ingredients S/A, which is located in Ananindeua/PA. Hyaluronic acid (Lot: PS009531/F01) was obtained from a Manipulation Pharmacy in Belém/PA, and the 12-hydroxystearic structuring agent (Lot: E8050A) was supplied by Alfa Aesar (Tewksbury, MA, USA).

### 4.2. Preparation of Organogel Systems

The acai oil-based organogel structured with 12-hydroxystearic acid (12-HSA) as gelling agent was prepared by weighing the equivalent of 2% (*m*/*m*) of the structuring agent (12-HSA) to 100% (*m*/*m*) of acai oil. The mixture was heated at 85 °C for 30 min in a water bath. Then, it was stirred for 3 min using a magnetic stirrer. Subsequently, the mixture was cooled to room temperature for 24 h in order to obtain the organogel. The same procedure was used to produce organogel (OG) without and with 1% (*m*/*m*) of hyaluronic acid incorporated in the mixture (OG + HA) before the heating phase [72]. The percentage mass compositions of OG and OG + HA formulations are given in Table 4.

### 4.3. Fourier-Transform Infrared Spectroscopy (FTIR)

OG and OG + HA samples were analyzed under the following conditions: scan 32, resolution 4 cm^−1^ and in the range 4000–600 cm^−1^ (Shimadzu Corporation IR Prestige 21 Cat. No. 206-73600-36- Kyoto-Japan^®^) [73].

### 4.4. Polarized Light Optical Microscopy (PLOM)

The organogels morphology (OG and OG + HA) was evaluated by using a polarized light microscope with 50× magnification coupled to a Nikon LV-UEPI digital video camera made in Japan with a digital image capture system associated with the software interconnected with the microscope [72].

### 4.5. Thermal Analysis

Thermogravimetry/derived thermogravimetry (TG/DTG) and differential scanning calorimetry (DSC) analyses of OG and OG + HA were performed under the following conditions: 5 mg of the samples added in platinum crucibles, nitrogen atmosphere (50 mL/min), and heating rate of 10 °C/min in a temperature range of 25 to 600 °C. The mass loss and enthalpy variation calculations were performed with the aid of TA-60WS software (Thermal Analysis Workstation Shimadzu, EUA^®^, Columbia, MD, USA) [74].

### 4.6. Texture Profile Analysis

A double compression test was performed on OG and OG + HA organogels in order to evaluate hardness, spreadability, stickiness, and gumminess by using a texturometer (Texture Analyser, TA-XT Plus, Surrey, UK) controlled by microcomputer. We used a cylindrical acrylic specimen of 25 mm in diameter and 35 mm in length, as well as speed of 1.0 mm/s and a fixed distance for specimen penetration of 15 mm. The value considered was the maximum force obtained, regardless of the depth of the specimen when measuring this value [72].

### 4.7. Rheology

Rheological analyzes were performed by using a controlled voltage rheometer (Physica, MCR 101, Ostfildern, Germany) with parallel plates with a rough surface (40 mm diameter and 200 μm gap). The temperature was controlled by using a Peltier system. The rheological properties of organogels were evaluated by low-amplitude oscillatory measurements by considering two rates (3 and 10 °C/min). In both conditions, the samples were heated to 80 °C and cooled to 15 °C. Subsequently, they were heated from 15 to 80 °C. A frequency (_) of 1 Hz and 1% of deformation were used. The sample was placed in the equipment and equilibrated for 5 min before the beginning of the analyses. Then, the temperature ramps were applied. The following were determined: elastic modulus (G′), viscous module (G″), complex module (G*), phase angle (tan(_)), and complex viscosity (_*). Determinations were performed in triplicate [72].

### 4.8. Quantification of Hyaluronic Acid

The analysis of the hyaluronic acid concentration from OG + HA formulation was performed by the modified carbazole method proposed by Bitter and Muir (1962), which breaks the bonds between the hyaluronic acid chains of the particles and binds with the released uronic acid groups [75]. A total of 0.1 g of OG + HA was dissolved in 20 mL 0.1 M phosphate buffer pH 7 under sonication. Then, the HA solution was further diluted in 100 mL final volume in 0.1 M phosphate buffer with pH 7 [76].

Initially, 3 mL of a 0.025 M solution of sodium tetraborate decahydrate (Na_2_B_4_O_7_·10H_2_O) in sulfuric acid was placed in neutral glass tubes arranged on a grid and cooled to 4 °C. Then, it was added to the sample (0.5 mL). The addition took place in an ice bath as it is a very exothermic reaction. The tubes were kept closed with aluminum foil and placed under constant agitation for complete mixing of the system. Each mixture was placed in a bath at 100 °C for 10 min. It was allowed to cool down to room temperature. Then, 0.1 mL of carbazole solution (0.125% in ethanol) was added with subsequent stirring. The solution was again placed in a bath at 100 °C for 15 min followed by the same process of cooling to room temperature. Finally, the sample was read in the spectrophotometer at 530 nm (1800 UV-VIS, Shimadzu Europe Analytical Instruments, USA^®^, Norwood, MA, USA), and the HA concentration was calculated from the equation of line obtained after validating the linearity of the method.

#### Validation Parameters

Linearity was calculated by the average of three intervals with authentic repetitions, which included six concentrations diluted from a 100 µg/mL hyaluronic acid solution (1.5, 2.5, 5, 7.5, 10.0, and 12.5 µg/mL) which were analyzed at ƛ of 530 nm in a spectrophotometer. After visual linear relationship, the results were statistically analyzed in order to define the coefficient of determination (minimum acceptable R^2^ = 0.99), regression equation, linear fit, and relative standard deviation. The mean linear regression equation obtained from the three calibration curves was y = 14.567x − 2.1142. The coefficient of determination obtained was R^2^ = 0.9965, which proves the adequacy of the method to the evaluated interval. The precision parameter was evaluated by repeatability by using samples at the same concentration (7.5 µg/mL) and on the same day (intra-run). For intermediate precision, samples at the same concentration (7.5 µg/mL) were also used. The assay was performed by two different analysts on two consecutive days (intra-run), both in sextuplicate. Accuracy was evaluated by three controls (in triplicate) of low (1.5 µg/mL), medium (7.5 µg/mL), and high (12.5 µg/mL) concentrations. Limits of detection (LD) and quantification (LQ) were estimated in µg/mL [77,78].

### 4.9. Oxidative Stability of Organogel

In the investigation of accelerated stability, 5 g of OG + HA was placed in a neutral, transparent, and hermetically sealed glass bottle. The flasks were stored in a refrigerator (2–8 °C) at room temperature (25–30 °C) and in a kiln (40–45 °C) for 90 days. The evaluated properties were organoleptic (color, odor, texture, and phase separation). The pH and concentration of hyaluronic acid were at time zero (24 h after preparation of organogel) at 7, 15, 30, 45, 60, and 90 days, according to the Cosmetic Products Stability Guide made available by ANVISA [79].

### 4.10. Data Analysis

The mean and the standard deviation (SD) were calculated. Statistical comparisons were made by using the analysis of variance (ANOVA, single factor) through GraphPad Prism™ software version 8.00 for Windows^®^ (GraphPad Software, San Diego, CA, USA). *p* < 0.0001 as the significance level was adopted.

## Figures and Tables

**Table 1:** Physicochemical characteristics of OG + HA stored at room temperature for 90 days.

Time (Days)							
Room (25–30 °C)	0	7	15	30	45	60	90
pH	6.9 + 0.03	6.7 + 0.01	6.1 + 0.012	6.3 + 0.005	5.9 + 0.002	6.0 + 0.05	5.7 + 0.004
Concentration HA (µg/mL)	11.22 + 0.001	6.02 + 0.013	8.92 + 0.003	7.61 + 0.030	5.19 + 0.01	6.11 + 0.002	6.61 + 0.003
Concentration HA (%)	100	60.80	90.10	76.86	52.42	61.71	66.76

**Table 2:** Physicochemical characteristics of OG + HA stored at refrigeration temperature for 90 days.

Time (Days)							
Refrigeration (2–8 °C)	0	7	15	30	45	60	90
pH	6.9 + 0.03	6.7 + 0.01	6.9 + 0.01	6.1 + 0.001	5.8 + 0.02	6.4 + 0.002	5.7 + 0.05
Concentration HA (µg/mL)	11.22 + 0.001	5.92 + 0.001	9.08 + 0.04	8.05 + 0.011	6.85 + 0.001	6.88 + 0.003	5.24 + 0.01
Concentration HA (%)	100	59.79	91.71	81.31	69.19	69.49	52.92

**Table 3:** Physicochemical characteristics of OG + HA stored at kiln temperature for 90 days.

Time (Days)							
Kiln (40–45 °C)	0	7	15	30	45	60	90
pH	6.9 + 0.03	6.5 + 0.04	6.2 + 0.02	5.7 + 0.05	5.5 + 0.001	6.9 + 0.01	5.8 + 0.06
Concentration HA (µg/mL)	11.22 + 0.001	8.31 + 0.001	7.89 + 0.012	7.76 + 0.003	5.81 + 0.01	6.62 + 0.02	5.82 + 0.001
Concentration HA (%)	100	83.93	79.69	78.38	58.68	66.86	58.78

**Table 4:** Composition of the organogel of acai oil.

Constituents of the Organogel	OG (% (*m*/*m*))	OG + HA (% (*m*/*m*))
Acai oil	100	100
12-HSA	2	2
Hyaluronic acid	-	1

## Data Availability

Data are contained within the article.
